# Supplementary material for: Bacillus Calmette-Guerin Infection in NADPH Oxidase Deficiency: Defective Mycobacterial Sequestration and Granuloma Formation
Source: PLoS Pathog. 2014 Sep 4;10(9):e1004325. doi: 10.1371/journal.ppat.1004325 (PMC4154868; doi:10.1371/journal.ppat.1004325)
Supplement: Figure S1 — Similar phenotypes in naïve mice. (A) Lung, liver and spleen histology (Hematoxylin and eosin staining) from wild-type, Ncf1 mutant and Ncf1 rescue mice without BCG infection. (B) Organ weight related to body weight of wild-type (n = 3), Ncf1 mutant (n = 3) and Ncf1 rescue (n = 3) mice without BCG infection. Lung weight ratio was in figure 4A. Magnifications were ×100. (PPT) [file ppat.1004325.s001.ppt]

## Slide 1
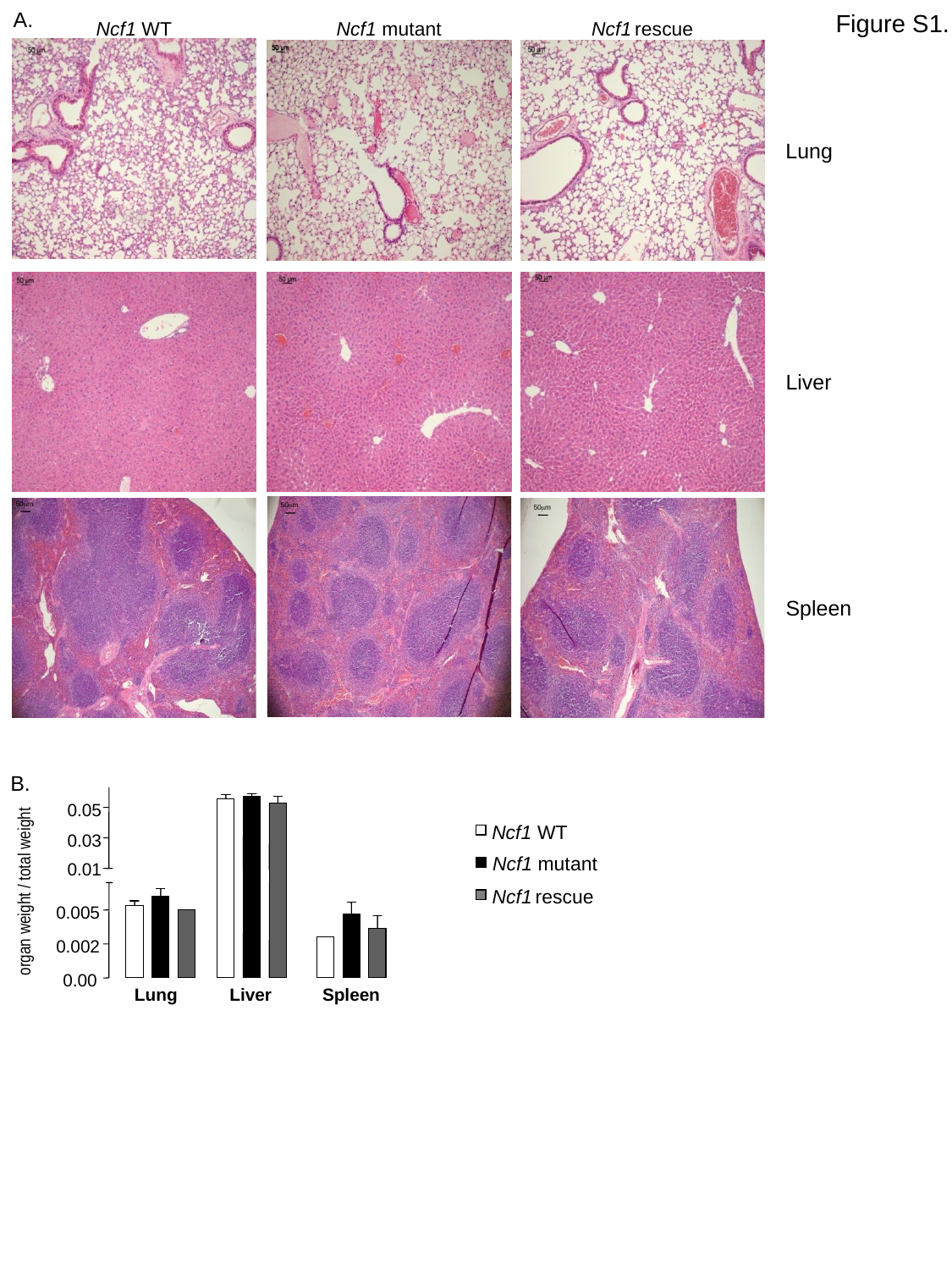

A.
Figure S1.
Ncf1 WT
Ncf1 mutant
Ncf1 rescue
Lung
Liver
50μm
50μm
50μm
Spleen
B.
Liver
0.05
0.03
0.01
organ weight / total weight
Lung
0.005
Spleen
0.002
0.00
Ncf1 WT
Ncf1 mutant
Ncf1 rescue
