# Supplementary figures and images for: Bacillus Calmette-Guerin Infection in NADPH Oxidase Deficiency: Defective Mycobacterial Sequestration and Granuloma Formation
Source: PLoS Pathog. 2014 Sep 4;10(9):e1004325. doi: 10.1371/journal.ppat.1004325 (PMC4154868; doi:10.1371/journal.ppat.1004325)

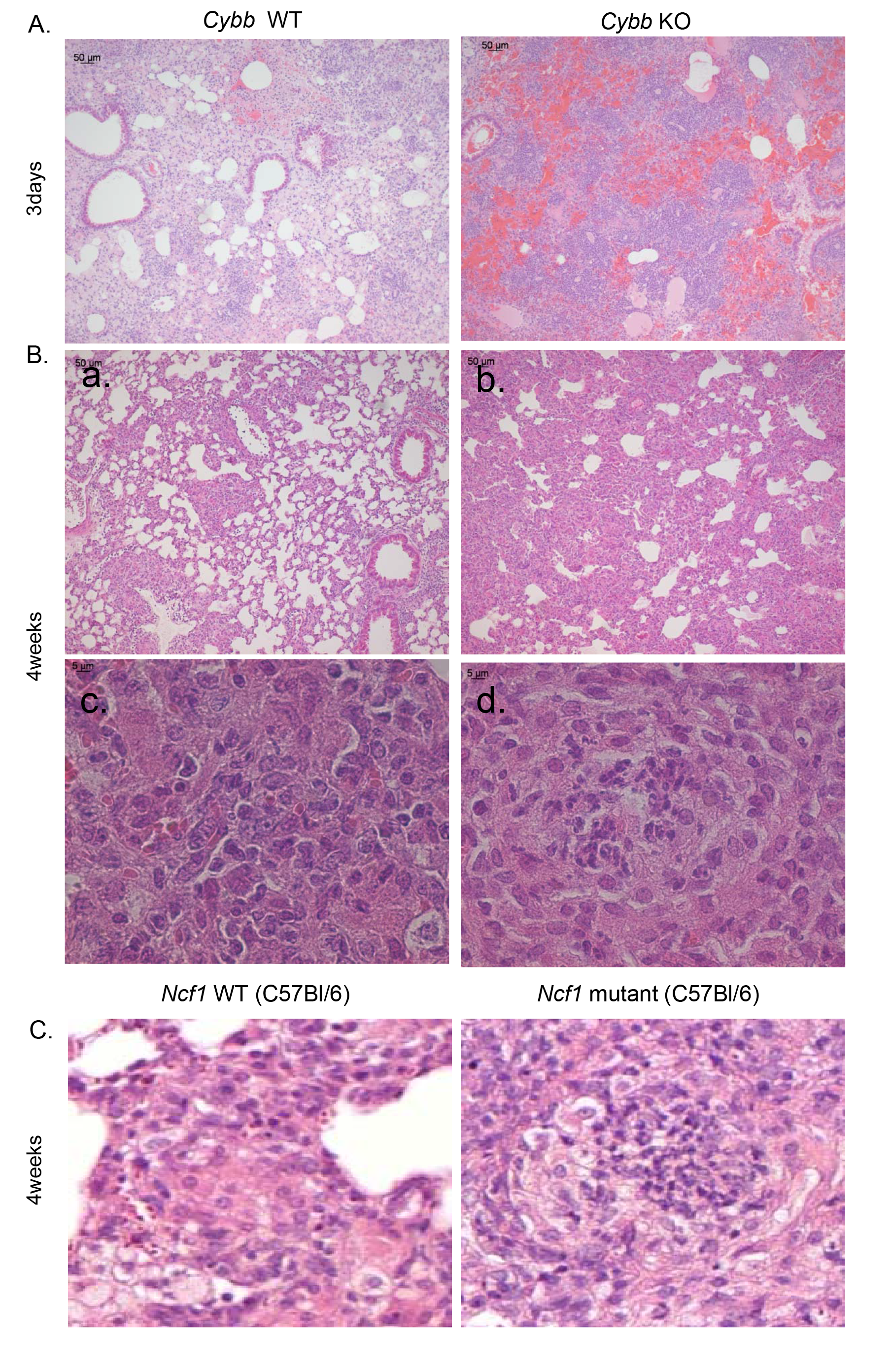

Supplement: Figure S2 — Lung damage in response to BCG infection in additional CGD mouse models. Lung histology (Hematoxylin and eosin staining) from Cybb wild-type (left panels) and Cybb -deficient (right panels) mice, 3 days (A) and 4 weeks (B) after BCG infection. Three days post-infection, hemorrhagic pneumonia was observed in Cybb -deficient mice. Four weeks after BCG infection, Cybb -deficient lungs show a massive inflammation, alveolar obstruction (B-b). Higher magnifications show massive infiltration of neutrophils only in Cybb -deficient lung (B-d). (C) Ncf1 mutant mice with C57Bl/6 genetic background show also abscess of neutrophils, absent in respective wild-type mice. Magnifications were ×100 and ×1000. (TIF) [file ppat.1004325.s002.tif]
