# Supplementary material for: Bacillus Calmette-Guerin Infection in NADPH Oxidase Deficiency: Defective Mycobacterial Sequestration and Granuloma Formation
Source: PLoS Pathog. 2014 Sep 4;10(9):e1004325. doi: 10.1371/journal.ppat.1004325 (PMC4154868; doi:10.1371/journal.ppat.1004325)
Supplement: Figure S3 — Ex-vivo restimulation of splenocytes from BCG infected Cybb -deficient and wild-type mice. TNF and NO (nitric oxide) levels were evaluated in culture supernatant from splenocytes incubated with 103 viable M. bovis BCG (A and C) or with antigens derived from M. bovis BCG (B and D). Values are shown as mean ± SEM (n = 4–5 mice per group, assayed in triplicate). * p<0.05 (PPT) [file ppat.1004325.s003.ppt]

## Slide 1
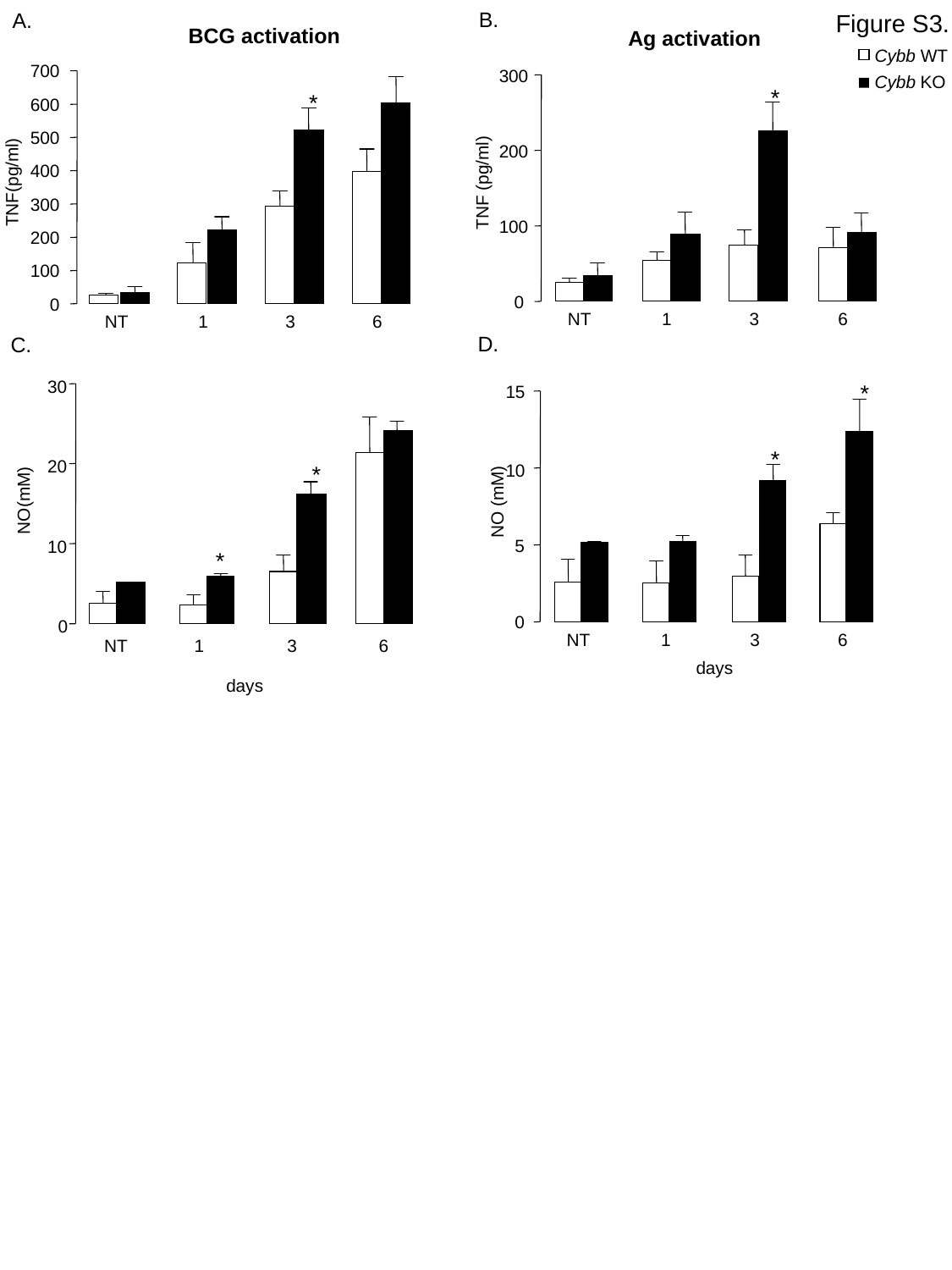

B.
A.
Figure S3.
BCG activation
700
*
600
500
400
TNF(pg/ml)
300
200
100
0
NT
1
3
6
Ag activation
Cybb WT
Cybb KO
300
200
TNF (pg/ml)
100
0
NT
1
3
6
*
D.
C.
30
*
15
*
20
10
*
NO(mM)
NO (mM)
5
10
*
0
0
NT
1
3
6
NT
1
3
6
days
days
