# Supplementary material for: Bacillus Calmette-Guerin Infection in NADPH Oxidase Deficiency: Defective Mycobacterial Sequestration and Granuloma Formation
Source: PLoS Pathog. 2014 Sep 4;10(9):e1004325. doi: 10.1371/journal.ppat.1004325 (PMC4154868; doi:10.1371/journal.ppat.1004325)
Supplement: Figure S4 — Granuloma phenotype in additional CGD mouse models. (A) Lung weight related to body weight of Ncf1 mutant with C57Bl/6 genetic background and Cybb -deficient mice, as well as their respective controls 4 weeks post infection (**p<0.01). (B) Representative hematoxylin and eosin (H&E) stained lung sections showing granulomas 4 weeks post infection. Magnifications were 200×. (PPT) [file ppat.1004325.s004.ppt]

## Slide 1
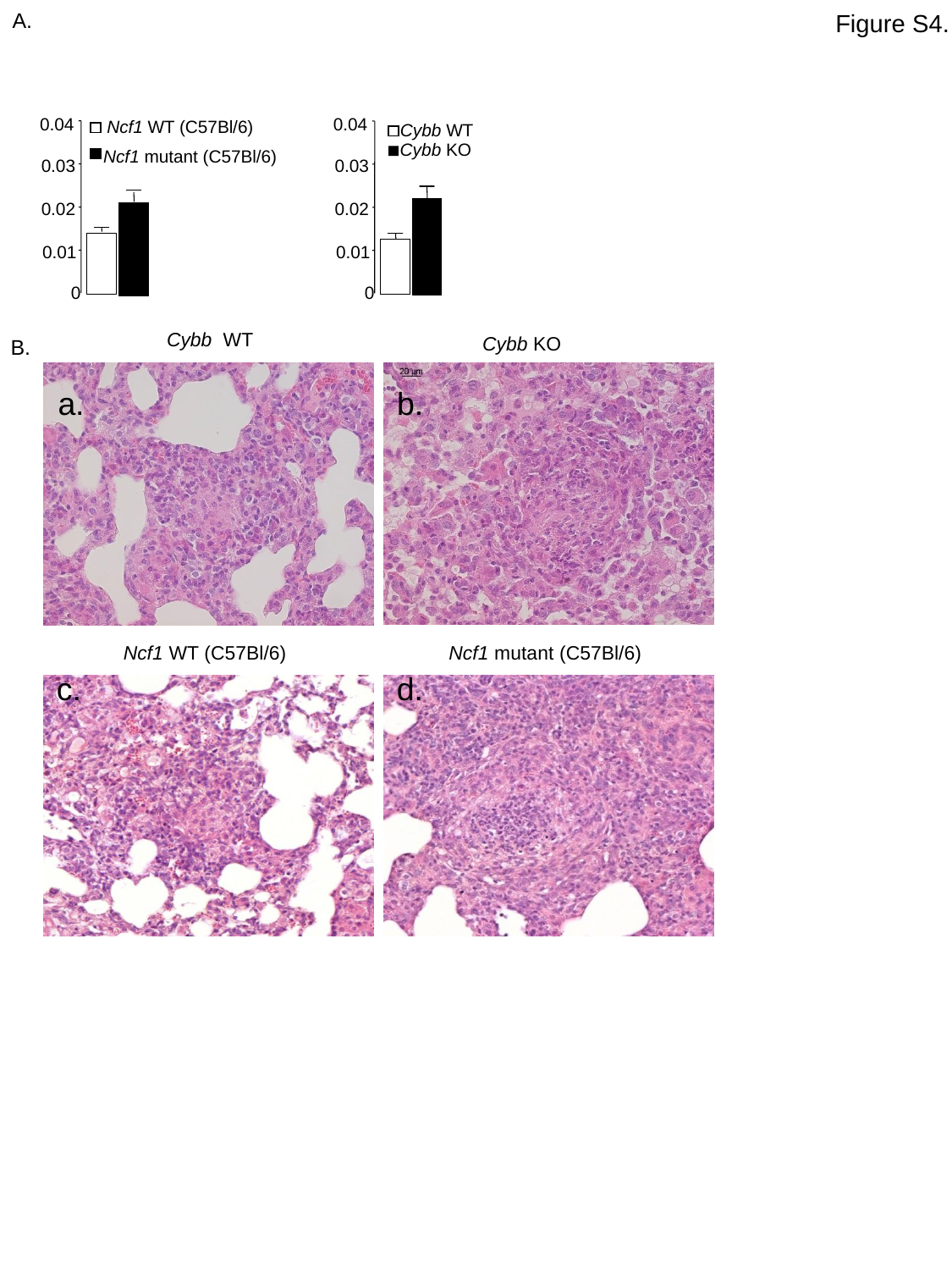

A.
Figure S4.
0.04
0.03
0.02
0.01
0
0.04
0.03
0.02
0.01
0
Ncf1 WT (C57Bl/6)
Ncf1 mutant (C57Bl/6)
Cybb WT
Cybb KO
B.
Cybb WT
Cybb KO
a.
b.
Ncf1 WT (C57Bl/6)
Ncf1 mutant (C57Bl/6)
c.
d.
